# Parents’ experiences of living with a child with cancer undergoing hematopoietic stem cell transplantation: a qualitative content analysis study

**DOI:** 10.3389/fpsyg.2024.1359978

**Published:** 2024-03-12

**Authors:** Maryam Maleki, Nahid Dehghan Nayeri, Amir Ali Hamidieh, Batool Pouraboli

**Affiliations:** ^1^Department of Pediatric and Neonatal Intensive Care Nursing Education, School of Nursing and Midwifery, Tehran University of Medical Sciences, Tehran, Iran; ^2^Nursing and Midwifery Care Research Center, School of Nursing and Midwifery, Tehran University of Medical Sciences, Tehran, Iran; ^3^Pediatric Cell and Gene Therapy Research Centre, Gene, Cell & Tissue Research Institute, Tehran University of Medical Sciences, Tehran, Iran

**Keywords:** experience, hematopoietic stem cell transplant, parent, pediatric oncology, qualitative study

## Abstract

**Objectives:**

Pediatric Hematopoietic Stem Cell Transplant (HSCT) profoundly impacts the physical, psychological, and social aspects of parents’ lives. Thus, this study aimed to explore the experiences of parents living with a child with cancer who undergoes HSCT.

**Methods:**

This qualitative study involved 20 parents of children with cancer who were undergoing HSCT at a referral hospital in Iran. Purposive sampling was used to select the participants from February 2023 to November 2023. In-depth semi-structured interviews, featuring open-ended questions, were utilized for data collection. Data analysis was performed using conventional content analysis.

**Results:**

Data analysis revealed two main themes. “Surrounded by hardships” and “Self-actualization.” The first theme encompassed participants’ experiences of facing difficulties in life after being aware of their child’s need for HSCT. This theme consisted of four categories: “uncertainty about the child’s future,” “exhaustion from the child’s treatment process,” “worrying about the healthy child(ren),” and “helplessness.” The second theme “self-actualization” included with two categories: “transformation in life’s philosophy” and “acquisition of new capabilities.” These categories highlighted the positive outcomes experienced by the participants following their child’s HSCT.

**Conclusion:**

Our findings underscore the importance of healthcare providers being attuned to parents’ experiences throughout their child’s HSCT trajectory. It is crucial for healthcare providers to encourage parents to articulate their concerns and feelings and seek support from healthcare providers, family, and friends. The development of psychological support services in healthcare settings can facilitate tailored interventions to alleviate parents’ difficulties.

## 1 Introduction

Pediatric cancer is identified as the second leading cause of death among children aged under 14 years (Faranoush et al., 2022). Globally, approximately 400,000 children (aged 0–19 years) receive a cancer diagnosis each year, with 70% being aged below the age of 15 years (World Health Organization, 2021; Deegan et al., 2023). Data from the American Cancer Society indicates that roughly 1 out of every 285 children aged below 20 is diagnosed with cancer. Iran is documented as having the sixth highest occurrence of childhood cancer in Asia, with an Age-Standardized Incidence Rate (ASR) of 13.6 per 100,000, as reported by the Global Cancer Observatory (Shabani et al., 2020), and the incidence rates stand at 140.9 and 150.7 per million for age groups 0–14 and 0–19, respectively (Steliarova-Foucher et al., 2017). A study conducted by Hashemi et al. (2024) on childhood and adolescent cancer in Iran revealed a standardized cancer incidence increase from 129.2 per million in 1999 to 132.3 per million in 2016 (Hashemi et al., 2024). Despite this daunting statistic, advances in cancer care and treatment over the past four decades have contributed to improved survival rates in pediatric oncology (Deegan et al., 2023).

Various factors including the utilization of advanced diagnostic techniques, enhanced supportive care, and therapeutic breakthroughs have contributed to the enhancement of the 5-year survival rate for childhood cancers to over 80% (Bhakta et al., 2019; Yeh et al., 2020). Among the therapeutic breakthroughs shaping this progress is Hematopoietic Stem Cell Transplantation (HSCT) (Pelletier et al., 2014).

For parents of children with cancer, HSCT often emerges as a crucial treatment option. Treatments such as conventional or standard chemotherapy treatments administered over a prolonged period may offer temporary disease control but there is a risk of disease progression and recurrence, which can potentially contribute to adverse outcomes. Consequently, despite the inherent risks of mortality and treatment failure associated with HSCT, a substantial majority of parents willingly consent to this procedure for their children (Pelletier et al., 2014).

HSCT stands out as a viable and acceptable yet intricate treatment method for both malignant and non-malignant disorders (Sürer Adanir et al., 2017; Phan et al., 2021; Qatawneh et al., 2021; Niederwieser et al., 2022). Currently, high-risk malignant diseases, characterized by recurrence or resistance, constitute approximately 65–70% of pediatric HSCT cases (Pelletier et al., 2014). More than 24,000 HSCTs are performed in the United States every year (Norkin and Wingard, 2017; Khaddour et al., 2022). Pediatric HSCT comprises approximately 17% of HSCTs (Zanato et al., 2017). Analysis of the European Society for Blood and Marrow Transplantation (EBMT) survey data reveals a consistent and steady rise in the annual numbers of HSCT since 1990 (Passweg et al., 2020). In 2021, across 125 specialized pediatric centers spanning 26 countries, the count of pediatric patients (those under 18 years old) marks a collective increase of +5.6% in the total number of transplants compared to the figures recorded in 2020 (Passweg et al., 2023). Notably, Iran has witnessed a steady increase in annual pediatric HSCTs since 2007. In Iran, pediatric HSCT began in 1991, initially serving both pediatric and adult patients in a shared ward. However, in 2007, separate units were established for pediatric and adult HSCT, with patients aged 15 or younger directed to the pediatric ward (Hamidieh et al., 2015). Although there are no accurate statistics on pediatric HSCTs in Iran, numerous pediatric HSCT centers now operate across Iran.

The treatment trajectory of children undergoing HSCT involves a protracted and intensive treatment process, encompassing immunosuppression, costly and extended hospitalizations (Liu et al., 2020), a spectrum of physical and psychosocial problems (Lindahl Norberg et al., 2014; Manookian et al., 2014), and Graft-versus-Host Disease (GvHD) (Phan et al., 2021). While certain side effects may dissipate within the initial post-treatment year, others endure, presenting ongoing challenges for years or even becoming permanent (Lindahl Norberg et al., 2014; Manookian et al., 2014). Therefore, despite the life-saving potential and diverse applications of HSCT, the process significantly impacts children’s health and quality of life (Beckmann et al., 2021).

The disruption in the child’s functioning becomes a source of emotional distress for parents (Lindahl Norberg et al., 2014; Aftyka et al., 2017). The arduous, prolonged, and stressful nature of HSCT influences the structure and dynamics of the family, as well as the quality of life (Phipps et al., 2005; Fife et al., 2017), and jeopardizing the psychological and emotional well-being of parents (Beckmann et al., 2021). Parents face complex conditions not only during the HSCT process but also post-discharge, contending with healthcare system challenges, adherence to medication regimens, stringent hygiene practices, the possibility of transplant rejection, and ongoing caregiving responsibilities (Kaziunas et al., 2016; Hoegy et al., 2019).

Research by Sürer Adanir et al. (2017) demonstrated elevated levels of obsessive-compulsive, anxiety, phobic anxiety, and interpersonal sensitivity in mothers with children undergoing HSCT compared to mothers with healthy children (Sürer Adanir et al., 2017). In addition, the findings of a qualitative study in China revealed parental concerns about the extended road to recovery, potential disease recurrence, managing childcare issues at home, and the possibility of chronic GvHD and infertility in their children undergoing HSCT (Liu et al., 2020). Additionally, a systematic review revealed that HSCT in children was associated with a substantial increase in anxiety, depression, distress, and post-traumatic stress disorder in their parents (Packman et al., 2010). Moreover, Coleman et al. (2018) identified significant sleep disturbances in parents of children undergoing HSCT (Coleman et al., 2018). A cross-sectional descriptive study reported that the mental health of parents of children undergoing HSCT was below the normal level (Buchbinder et al., 2019). Another descriptive study reported low global mental health scores and high levels of anxiety and depression in parents of surviving children undergoing HSCT in the United States (Ward et al., 2019).

Contrary to these distressing experiences, pediatric HSCT can instigate positive changes in parents’ lives (Chardon et al., 2021). While inherently distressing, the event prompts coping efforts, leading parents to construct a new worldview that allows for a positive reappraisal of HSCT over time (Tedeschi et al., 2018). Both a quantitative study by Riva et al. (2014) and a qualitative study by Beckmann et al. (2021) affirmed that parents often undergo positive changes following their child’s HSCT. Given that quantitative research examines the hypothesis irrespective of historical, social, and cultural contexts. Therefore, to comprehensively understand the experiences of parents of children undergoing HSCT, it is necessary to use qualitative methods. Qualitative research enables the exploration of attitudes and behaviors and offers crucial insights into parents’ experiences (Shetgiri et al., 2015).

Exploring these experiences can inform the design of interventions aimed at enhancing parental quality of life and mental health. While some literature has discussed the challenges faced by parents of children undergoing HSCT, these experiences vary according to contextual factors. Hence, further research across diverse countries is warranted. Iran, as an Islamic nation with a prevailing religious culture (Farsi et al., 2010), may yield experiences among parents distinct from those in other cultural settings. Moreover, to our knowledge, only one qualitative study has explored the experiences of six parents with children undergoing bone marrow transplantation (BMT) for both malignant and non-malignant diseases in Iran, conducted in 2011 (Asadi et al., 2011). In general, the experiences of parents dealing with child’s HSCT multifaceted, encompassing various psychological, cultural, familial, religious, and existential elements (Cavadini et al., 2019). Additionally, the experiences of parents whose children are undergoing HSCT for cancer may differ significantly from those whose children are undergoing HSCT for non-malignant diseases. Therefore, by narrowing our focus to children with cancer, we could better understand the specific challenges and psychosocial dynamics associated with this population. Hence, given Iran’s distinct cultural attributes, the nature of family relationships, the traditional societal fabric, and its emphasis on family-centered values, this study was conducted to explore the parents’ experiences of living with a child with cancer undergoing HSCT.

## 2 Methods

### 2.1 Design and participants

This qualitative study employed a conventional content analysis approach. The content analysis approach facilitates the systematic coding of textual data for description and interpretation (Vaismoradi and Snelgrove, 2019). The study was carried out at the Children’s Medical Center, affiliated with Tehran University of Medical Sciences, Tehran, recognized as the largest pediatric HSCT center in Iran, spanning from February 2023 to November 2023. This facility as one of the pioneering centers performs approximately 100 pediatrics HSCTs annually.

Inclusion criteria comprised parents of children undergoing HSCT for cancer, proficient in Persian, capable of expressing their experiences, and willing to participate in the research. Parents were excluded if they were diagnosed with a mental illness, had a child undergoing HSCT for a non-malignant disease, or experienced the death of a child during HSCT.

### 2.2 Ethical considerations

The study protocol received approval from the Ethics Committee affiliated with Tehran University of Medical Sciences (decree number: IR.TUMS.CHMC.REC.1401.145). Permissions to access participants’ information were obtained, and participants were informed about the study’s purpose, voluntary participation, data confidentiality, the right to withdraw at any point, and anonymity. Informed consent and permission to audio record interviews were obtained from participants before each interview.

### 2.3 Data collection

Purposive sampling was employed, with the first participant selected through consultation with nurses familiar with the parents. Subsequent participants were identified through the review of medical files by the first author (MM). Face-to-face meetings during medical visits or phone calls were used to invite eligible parents for interviews. Interviews were conducted in Farsi at the hospital or participants’ homes by the first author (MM).

Firstly, unstructured interviews and then in-depth semi-structured interviews with open questions were used. The unstructured interviews were conducted as an initial phase to allow participants to freely start with whatever they liked about the topic and express their thoughts and experiences without constraints. This approach aimed to build trust and create a comfortable environment for participants, ultimately facilitating deeper insights into their experiences during the subsequent semi-structured interviews. The interview guide included the following questions: (1) Talk about your experiences and feelings with your child’s HSCT (2). What changes happened in your life after the HSCT of your child? The depth of interviews was improved by asking probing questions as follows: Can you talk about it in more detail? Can you provide an example? Interviews lasted between 40 and 60 min. Sampling continued until data saturation, evidenced by the development of categories and subcategories without the introduction of new findings through additional interviews. A total of 20 participants were included in this study. Additionally, a sociodemographic questionnaire collected information on participants’ age, number of children, education level, economic status, occupation, place of residence, and details about the child, including age, gender, type of cancer, birth rank, type of HSCT, and duration after HSCT.

### 2.4 Data analysis

The first author (MM) transcribed the interviews verbatim, and the research team conducted data analysis using the content analysis approach proposed by Graneheim and Lundman (2004) and Vaismoradi et al. (2013). Multiple readings of transcribed interviews were performed to gain a rich understanding of the collected data. Meaning units were identified, and related codes were assigned. Codes were then organized into categories and subcategories based on similarities and differences (Vaismoradi et al., 2013; Vaismoradi and Snelgrove, 2019).

### 2.5 Trustworthiness of data

Lincoln and Guba’s criteria—credibility, transferability, dependability, conformability, and authenticity—were employed to enhance the trustworthiness of the data (Polit and Beck, 2009; Thomas and Magilvy, 2011). Prolonged engagement, peer and member checking, and maximum variance in participant selection contributed to credibility. A summary of findings was provided to some participants for member checking, ensuring the research results reflected their perspectives. Peer checking involved approval of codes, categories, and subcategories by two qualitative researchers. Initial coding was done by the first author. Then it was checked by two other researchers. Any discrepancies were resolved through discussion with team members and consensus. Furthermore, considering the inclusion criteria, participants were selected with maximum variation in terms of age, education level, occupation, economic status, number of children, time passed from child’s HSCT, and type of child’s HSCT. The study’s transferability was ensured through a comprehensive explanation of participants, context, and findings, allowing readers to relate the study to their own contexts. A detailed description of the study’s purpose and research processes was provided to establish the dependability of the study.

## 3 Results

The participants had an age range of 26–53 years. Most of the parents were mothers (60%), with 60% having two children and 50% possessing an academic education degree. Regarding their children, the majority were girls (60%), held the first birth rank (50%), and were aged between 4 and 21 years. Additionally, 55% of the children received a diagnosis of Acute Lymphocytic Leukemia (ALL), and 65% underwent allogeneic HSCT (Tables 1, 2).

**Table 1:** The demographic characteristics of parents.

Variable	*N*
*Age, range (y)*	26–53
*Participant*	*n* (%)
Mother	12 (60)
Father	8 (40)
*Education level*	
Under diploma	5 (25)
Diploma	5 (25)
Academic	10 (50)
*Place of residence*	
City	18 (90)
Village	2 (10)
*Number of children*	
One child	4 (20)
Two children	12 (60)
Three or more children	4 (20)
*Occupation*	
Employed	10 (50)
Housekeeper	10 (50)
*Economic status (self-report)*	
Sufficient	2 (10)
Relatively sufficient	6 (30)
Not sufficient	12 (60)

**Table 2:** The demographic characteristics of children.

Variable	
*Age, range (y)*	4–21
*Gender*	*n* (%)
Girl	12 (60)
Boy	8 (40)
*Birth rank*	
First	10 (50)
Second	8 (40)
Third or more	2 (10)
*Type of cancer*	
ALL	11 (55)
Neuroblastoma	4 (20)
Wilms tumor	3 (15)
AML	2 (10)
*Type of HSCT*	
Autologous	7 (35)
Allogeneic	13 (65)
*Time passed from HSCT*	
< 1 year	13 (65)
1–5 years	5 (25)
> 5 years-10 years and more	2 (10)

The participants underwent significant changes in the aftermath of their child’s cancer-related HSCT. Through meticulous data analysis, we identified a total of 631 meaning units, which were subsequently categorized and coded. Finally, 521 codes were developed based on these meaning units after merging duplicate codes. Then, we identified 18 subcategories, classified into six categories, ultimately converging into two main themes. The study’s findings are detailed in Table 3.

**Table 3:** Subcategories, categories, and themes developed in this study.

Subcategory	Category	Theme
Worrying about disease recurrence in the child Conflicting emotions about HSCT Mental distress from the potential rejection of the child’s HSCT Fear of losing the child Concerns about the child’s future	Uncertainty about the child’s future	Surrounded by hardships
Stressful feelings related to invasive procedures Annoying side effects of treatments Mental distress associated with the results of diagnostic procedures	Exhaustion from the child’s treatment process	
Worrying about the donor’s healthy child Worrying about the non-donor healthy child (ren)	Worrying about the healthy child (ren)	
Feelings of aloneness Physical burnout Emotional fatigue	Helplessness	
Gain new insights Willingness to help	Transformation in life’s philosophy	Self-Actualization
Achieving new personal power Rebuilding relationships Spiritual growth	Acquisition of new capabilities	

### 3.1 Surrounded by hardships

HSCT cast a pervasive shadow over nearly every aspect of parents’ lives. Upon becoming aware of their child’s necessity for HSCT, participants encountered numerous challenges. The main theme, “surrounded by hardships” encompasses four categories: “uncertainty about the child’s future,” “exhaustion from the child’s treatment process,” “worrying about the healthy child(ren),” and “helplessness.”

#### 3.1.1 Uncertainty about the child’s future

Practically all participants voiced uncertainties regarding their child’s future, encompassing fears of disease relapse, rejection of HSCT, potential loss of their child, and the trajectory of their child’s life post-HSCT. Within this category, five subcategories emerged: “worrying about disease recurrence in the child,” “conflicting emotions about HSCT,” “mental distress from the potential rejection of the child’s HSCT,” “fear of losing the child,” and “concerns about the child’s future.”

##### 3.1.1.1 Worrying about disease recurrence in the child

The prospect of disease recurrence, both before and after HSCT, instilled fear, discomfort, anxiety, and stress among the participants. A participant vividly expressed his concerns about the future, stating:

*“I am very worried about his future, and I do not know what will happen in the future. What if his illness relapses?” (Participant (P)8, Father).*

The emergence of physical symptoms in the child heightened worries about disease recurrence. Several participants attributed their anxiety to factors such as opting for autologous HSCT and receiving insufficient explanations from doctors regarding suspicious scan results:

*“Since we are using his own bone marrow, we are afraid of relapse. But those who receive it from someone else have a much better chance of recovery” (P9, Father).*

##### 3.1.1.2 Conflicting emotions about HSCT

Participants grappled with conflicting emotions concerning HSCT. On one hand, they found solace and happiness in the existence of a viable treatment option for their child, recognizing the potential for successful treatment. On the other hand, they expressed feelings of stress and apprehension associated with HSCT due to lingering doubts about its consequences:

*“I kept telling myself that it’s a relief that there’s a suitable treatment for my child’s illness and that he’ll get better. I was very happy about that. But at the same time, I was worried because the transplant came with many challenges, and all of it was concerning to me” (P11, Mother).*

##### 3.1.1.3 Mental distress from the potential rejection of the child’s HSCT

Parents consistently expressed their fear, stress, anxiety, and mental preoccupation regarding the potential rejection of their child’s HSCT, both before, during, and after the procedure. The majority experienced this fear due to various factors, including the necessity for their child to undergo a second injection of stem cells, witnessing instances of HSCT rejection in other children, concerns about the development of GvHD, and their child contracting COVID-19:

*“I believe it was about 2 months after her transplant when they (physicians and nurses) mentioned she needed another cell injection. We were anxious because we thought her transplant had definitely been rejected” (P4, Mother).*

Some parents conveyed a perpetual and enduring concern about the potential rejection of their child’s HSCT. They associated minor changes in their child’s physical condition with the looming possibility of HSCT rejection:

*“A few days after leaving the hospital, we suddenly noticed that my child’s skin had become scaly. We were all concerned that his transplant might have been rejected again” (P3, Mother).*

##### 3.1.1.4 Fear of losing the child

The participants expressed feelings of fear, stress, anxiety, worry, and mental preoccupation regarding the potential loss of their child. This apprehension stemmed from concerns about the recurrence of the disease, the necessity for intensive chemotherapy, the possibility of complications arising from chemotherapy and HSCT, receiving news about the demise of children undergoing HSCT, and witnessing the equipment used for cell harvesting:

*“When I entered the transplant department, I felt intense fear and stress. Because two out of three friends who had their children undergo transplant before us tragically lost their kids during the transplant process. I was afraid of the possibility of losing my own child” (P10, Mother).*

Moreover, the fear of losing the child was heightened among parents due to the COVID-19 pandemic:

*“We were worried that since he was undergoing intense chemotherapy, if he were to contract COVID-19, we might lose him” (P9, Father).*

##### 3.1.1.5 Concerns about the child’s future

The prolonged process of HSCT and the necessity for isolation hindered children undergoing HSCT from attending school and pursuing their education. Consequently, parents developed concerns about their child’s future education and career post-HSCT. Additionally, parents expressed significant worries about their children’s prospects of getting married and having children in the future:

*“My daughter has not been able to attend school for 2 years now, so I’m concerned about her education. Additionally, I worry that if she grows up and wants to get married, she may face fertility issues” (P17, Mother).*

#### 3.1.2 Exhaustion from the child’s treatment process

The process of the child undergoing HSCT encompasses phases before, during, and after the transplant. Depending on the nature of each of these phases, parents encountered challenges that gave rise to the category of “exhaustion from the child’s treatment process.” This category comprised three subcategories: “stressful feelings related to invasive procedures,” “annoying side effects of treatments,” and “mental distress associated with the results of diagnostic procedures.”

##### 3.1.2.1 Stressful feelings related to invasive procedures

From the diagnosis of cancer onward and throughout the HSCT process, children undergo numerous invasive diagnostic and therapeutic procedures. These procedures include intravenous line placement, port placement, chemotherapy, Intrathecal (IT) treatments, Central Venous line (CV line) placement, Bone Marrow Aspiration (BMA), and the injection of hematopoietic stem cells. As these procedures were challenging and distressing for children, parents, in turn, grappled with a multitude of stressful feelings, including discomfort, sadness, worry, fear, stress, and anxiety:

*“To obtain a bone marrow sample, they (physician and nurses) administered her anesthesia and gave her an injection. My child was in pain. She cried while the cells were being extracted, and I was deeply upset to see her like that” (P18, Mother).*

##### 3.1.2.2 Annoying side effects of treatments

Any treatment process may entail side effects, and HSCT is no exception. Participants reported various side effects experienced by their children during HSCT, encompassing the effects of preparatory chemotherapy such as mouth sores, vomiting, hair loss, darkening of the skin, pain, and suffering. Additionally, they noted side effects from medications, mood changes, aggression, as well as complications like pulmonary, respiratory, bladder, and brain GvHD. Children also faced challenges such as contracting COVID-19 and shingles due to a compromised immune system, and convulsions. These complications in children elicited worry, discomfort, fear, and mental preoccupation among the participants. Furthermore, parents expressed concerns about potential cell loss post-HSCT, unforeseen issues after discharge, and the risk of infection:

*“Prior to the transplant, he underwent a series of intense chemotherapy sessions, which were quite harsh. He experienced severe side effects, such as continuous vomiting of blood and darkening of the skin, which distressed me greatly” (P13, Mother).*

##### 3.1.2.3 Mental distress associated with the results of diagnostic procedures

The participants conveyed feelings of worry, stress, mental preoccupation, and anxiety while awaiting the results of their child’s diagnostic measures, including White Blood Cells (WBC) tests, Human Leukocyte Antigens (HLA) tests, infection tests during the post-transplant period, bone marrow assessments to determine contamination or transplant percentage, and Metaiodo benzylguanidine (MIBG) scans. The interval of anticipation for these diagnostic results emerged as a period of mental distress for the participants:

*“When those cells were injected, my mind was preoccupied with what the results of the samples would be. Every day, I anxiously awaited the test results for my child” (P5, Father).*

#### 3.1.3 Worrying about the healthy child(ren)

Beyond the child undergoing HSCT, the majority of participants had additional children, giving rise to heightened concerns. Their worrying about the healthy child(ren) manifested as both “worrying about the donor’s healthy child” and “worrying about the non-donor healthy child(ren).”

##### 3.1.3.1 Worrying about the donor’s healthy child

Some children underwent autologous HSCT, while others received allogeneic HSCT. Parents whose child required allogeneic HSCT and had another child as the donor expressed additional concerns. These apprehensions included worry, stress, and fear regarding the health status of the donor child, the apheresis process, the necessity for CVline placement for apheresis, and the potential complications such as seizures and anemia in the donor’s child. This concern was more pronounced for participants with a younger donor child, as in certain instances, it was necessary to retake cells to meet the required cell threshold for the child undergoing HSCT:

*“I felt conflicted. On one hand, I was happy that my other child was a donor, but on the other hand, I was sad and afraid that this child would also face problems” (P6, Mother).*

Moreover, some participants reported a conflicted sentiment concerning the utilization of umbilical cord blood cells from a healthy child for the HSCT of a sick sibling. While they found solace in the potential lifesaving role of the stored umbilical cord blood cells for the ailing child, they grappled with feelings of injustice and guilt towards the healthy child. This unease stemmed from worries and fears that the healthy child might require the stored cells in the future. Consequently, this concern for the well-being of the donor’s healthy child was so profound that some parents wished to be the donor themselves:

*“I thought to myself, ‘Well, my daughter has this problem. Now if we transplant my son’s umbilical cord cells to her, what will happen if my son has a problem in the future and needs these cells?”(P12, Father).*

##### 3.1.3.2 Worrying about the non-donor healthy child(ren)

The period encompassing a child’s HSCT is prolonged, demanding the constant presence and care of the parents, often involving periods of quarantine. Consequently, parents invest a significant amount of time in tending to their child undergoing HSCT. However, this commitment restricts the opportunity for parents to be present with their healthy children. Hence, one of the primary concerns voiced by participants centered around their non-donor healthy child(ren). Worries encompassed feelings of loneliness, physical distance, and uncertainties regarding the educational future of the other child. This concern was particularly amplified for participants whose healthy child was an infant, as they had to navigate leaving their child during a period requiring heightened care. Consequently, these parents felt a sense of falling short in fulfilling their parental responsibilities towards their other child(ren), expressing sentiments of guilt and inadequacy:

*“All I worry about is my daughter, who stays in our own city. When my husband comes to visit us, she stays at home alone. I am a mother, and I always worry about her” (P3, Mother).*

#### 3.1.4 Helplessness

Parents grappled with a sense of helplessness, stemming from the numerous challenges and obstacles encountered throughout their child’s HSCT. This sense of helplessness encompassed “feelings of aloneness,” “physical burnout,” and “emotional fatigue.”

##### 3.1.4.1 Feelings of aloneness

The majority of participants hailed from distant cities and regions and traveled to the HSCT center to follow up their child’s treatment process. Given the limited number of HSCT centers for children in Iran, concentrated in a few large provinces, parents faced the necessity of extensive travel. Additionally, stringent health protocols aimed at preventing infection in the child undergoing HSCT required participants to curtail their interactions with relatives and friends. Consequently, they grappled with feelings of isolation, loneliness, and homesickness, characterizing these emotions as challenging and uncomfortable:

*“We came from our city to the capital for the transplant, and I did not go back for 5 years and I did not see our relatives, and it was very difficult for me to be away from my family” (P10, Mother).*

##### 3.1.4.2 Physical burnout

Participants encountered numerous challenges throughout their child’s HSCT, encompassing various physical issues such as weakness, fatigue, hair loss, dizziness, weight loss, hypertension, hypothyroidism, diabetes, and sleep disorders. These physical issues led to their physical burnout:

*“I did not have any problems before my son’s transplant, but after that, I experienced insomnia, hypertension, and hypothyroidism”(P20, Mother).*

##### 3.1.4.3 Emotional fatigue

Emotional fatigue constituted another distressing consequence that participants underwent in the aftermath of their child’s HSCT. Throughout this period, participants articulated experiences of inner chaos, diminished morale, difficulty concentrating, feelings of depression and obsession, contemplation of suicide, and an inability to effectively cope with their child’s challenges:

*“There were days when I even contemplated suicide. I would tell myself it was my fault. I was stressed, I was greedy, and I thought somehow that I was the cause of my child’s illness” (P10, Mother).*

### 3.2 Self-actualization

Despite facing challenges in their lives, participants also derived significant positive outcomes following their child’s HSCT. These positive consequences were categorized under “transformation in life’s philosophy” and “acquisition of new capabilities”:

#### 3.2.1 Transformation in life’s philosophy

This category describes the participants’ experiences regarding the transformation they felt in their philosophy and worldview after their child’s HSCT and includes “gaining new insight” and “willingness to help.”

##### 3.2.1.1 Gaining new insight

The majority of participants expressed that they acquired a new perspective on life after their child’s HSCT experience. Some of them chose to adopt a less stringent and sensitive approach to everyday issues, seeking a more peaceful life:

*“I used to be quite sensitive, but not anymore. Now, I realize that life can change and end in an instant for anyone, so I’ve learned to stay calm” (P5, Father).*

Some participants experienced a transformation in their outlook towards life after their child’s HSCT. They reported a desire to be good and appreciate life after their child’s HSCT:

*“After the transplant, I’ve come to appreciate my child’s worth even more. I’ve gained a deeper understanding of the value of life; I believe these challenges are necessary for us to truly appreciate life” (P20, Mother).*

##### 3.2.1.2 Willingness to help

Throughout their child’s HSCT, parents received substantial financial support from a Non-Governmental Organization (NGO) dedicated to assisting children with cancer. Consequently, post-HSCT, they integrated a commitment to assisting others into their life philosophy. For instance, parents expressed a strong desire to contribute to this institution to extend financial aid to parents of children with cancer undergoing HSCT:

*“We used to provide minimal assistance to this (NGO) institution, but now, after my child’s transplant, we contribute more. We’ve come to realize how much it truly helps the patients. They pay for the medicine and hotels for people/families traveling from distant/other cities, and provide many other aids” (P6, Mother).*

Some participants extended their assistance to impoverished individuals or special patients. Furthermore, they volunteered as cell donors for other children in need of HSCT:

*“After my son’s transplant, I handed over the test results of myself, my wife, and my children to the hospital staff responsible for finding donors. I told them to contact us if our test results matched those of a child in need of a transplant” (P19, Father).*

#### 3.2.2 Acquisition of new capabilities

The participants reported acquiring new capabilities following their child’s HSCT. They experienced deep spirituality, felt stronger, and improved relationships with others. This category included three subcategories: “achieving new personal power,” “rebuilding relationships” and “spiritual growth.”

##### 3.2.2.1 Achieving new personal power

The participants noted that following their child’s HSCT, they became stronger in facing challenges. They gained renewed hope, optimism, and wisdom in their personal lives:

*"After the transplant, I felt a newfound strength within me. Now, no matter what challenges come my way, I'm determined to fight until the end" (P5, Father).*

Some participants highlighted the acquisition of experience and knowledge about HSCT:

*"With my child's transplant, I've gained valuable experience about the disease and the transplant process, enabling me to offer help and guidance to people in need" (P15, Father).*

##### 3.2.2.2 Rebuilding relationships

The participants recounted positive outcomes following their children’s HSCT, including a sense of peace and reconciliation with relatives. They also reported improved relationships with spouses and others, contributing to an overall improvement in their life situation compared to before:

*"I was divorced from my husband, but due to my daughter's plea, urging me to reconcile with her father for the sake of her illness, I had to remarry him in order to facilitate my daughter's treatment. This circumstance led my husband and me to live together again" (P17, Mother).*

##### 3.2.2.3 Spiritual growth

After their child’s HSCT, most participants underwent spiritual growth. They discussed the strengthening of their faith in God, sensing God in their personal lives, and feeling God’s presence as a constant companion:

*"After the transplantation, I concluded that the only constant companion of a person is God, and my faith in God has multiplied. I feel that God is more attentive to us now" (P11, Mother).*

Furthermore, some participants considered their child’s disease as a divine test:

*“My child's disease was a test from God, and I felt God in every moment of my child's disease and transplantation” (P12, Father).*

## 4 Discussion

This study provided a unique perspective on the parental experiences of living with a child undergoing HSCT for cancer. Participants felt surrounded by hardships due to uncertainty about their child’s future, exhaustion from the child’s treatment process, worrying about the healthy child(ren), and a sense of helplessness.

The uncertainty about child’s future stemmed from concerns such as worrying about disease recurrence in the child, experiencing conflicting feelings about HSCT, grappling with mental distress arising from the possibility of the child’s HSCT rejection, fearing the loss of the child, and harboring concerns about the child’s future. Similarly, a phenomenological study conducted in Iran, focusing on parents whose children underwent BMT, highlighted that a primary concern for parents revolved around the potential rejection of the transplant, disease recurrence, and the fear of losing their child (Asadi et al., 2011). Another qualitative study conducted by Liu et al. (2020) in China revealed that a main concern among parents of children undergoing HSCT included worries about transplant rejection, disease recurrence, chronic GvHD, and potential infertility in their child (Liu et al., 2020). Moreover, parents of children with primary immunodeficiency undergoing HSCT shared an essential experience characterized by uncertainty and the looming threat to their child’s life posed by both the disease and the treatment process (McdDowell et al., 2010). Moreover, as indicated by the findings of a qualitative study, the fear of transplant rejection and disease recurrence emerged as significant concerns that persisted in parents even one year after their child’s HSCT (Cavadini et al., 2019). Iran’s emphasis on familial relationships and the collective well-being of the family unit underscores the profound impact that a child’s illness can have on parents and their broader social networks. Moreover, societal expectations regarding parental roles and responsibilities may shape how parents navigate the uncertainties of HSCT treatment (Moghaddasi et al., 2018).

In light of these cultural and contextual factors, the findings from our study, along with those from related research conducted in Iran and other countries, underscore the universal nature of parental concerns during HSCT treatment. However, the specific nuances of these concerns within the Iranian cultural context highlight the importance of culturally sensitive and contextually tailored support for parents and families facing similar challenges. Recognizing and addressing these cultural dynamics is essential for providing comprehensive care and support to parents throughout the HSCT trajectory.

Exhaustion from the child’s treatment process manifested through the experience of stressful feelings related to invasive procedures, the burden of annoying side effects from treatments, and mental distress associated with the results of diagnostic procedures. Similar experiences are echoed in the literature concerning the exhausting trajectory of a child’s HSCT for parents. In a qualitative study aimed at elucidating parents’ experiences with HSCT in children with sickle cell anemia in France, all parents expressed a notable level of anxiety spanning the period before, during, and after their child’s hospitalization. This anxiety stemmed from fears of HSCT rejection, apprehensions about side effects, observations of physical changes in their child, such as alopecia and alterations in skin color, the challenges associated with procedures like anesthesia for BMA, and the separation of the child from the family (Cavadini et al., 2019). Moreover, another qualitative study revealed that parents viewed the transplant as a ray of hope, despite being aware of the potential threat to their child’s life (Forinder, 2004). This aligns with our research findings. Consequently, it becomes crucial to explore these sentiments in parents of children undergoing HSCT to identify vulnerable individuals and offer timely psychological support. This underscores the practical significance of the study’s findings. In Iran, where familial bonds and collective support are highly valued, parents often shoulder the emotional and physical burdens of their child’s illness and treatment process. Therefore, the exhaustion experienced by parents is not only a result of medical interventions but also reflects the weight of societal expectations and cultural norms surrounding caregiving and parental roles (Moghaddasi et al., 2018). The literature cited highlights the universality of parental experiences during a child’s HSCT trajectory, transcending cultural and geographic boundaries. However, within the Iranian context, where cultural, religious, and societal factors intersect, the exhaustion experienced by parents takes on unique dimensions.

Our findings revealed that another concern for parents of children undergoing HSCT was related to healthy donor children and healthy non-donor children. Consistent with our results, a qualitative study conducted by Asadi et al. (2011) on parents of children undergoing BMT found that participants experienced anxiety and stress regarding their other children. Similarly, in another study, parents expressed significant worry about the donor child, particularly when donors were young (Hutt et al., 2015). Given the cultural significance of family cohesion and the welfare of all family members in Iran, healthcare providers must recognize and address parents’ apprehensions regarding their other children during the HSCT process. Providing thorough information about the donation process, including potential risks and long-term implications, can empower parents to make informed decisions while alleviating their concerns.

The study’s findings showed that participants experienced helplessness characterized by feelings of loneliness, physical burnout, and emotional fatigue. Our findings align with the results of previous studies that highlighted the sense of isolation and loneliness experienced by parents following HSCT for their children (McdDowell et al., 2010; Cavadini et al., 2019; Beckmann et al., 2021). Another study found that HSCT in children reduced the emotional functioning of their parents (Terrin et al., 2013). Parents of children undergoing HSCT must participate in medical care while fulfilling their parental role, facing other chronic stressors such as frequent visits to doctors, lifelong follow-up after HSCT, late side effects of HSCT, work attendance, continuous financial burden, ongoing social participation, ongoing health issues, and maintaining family relationships (Buchbinder et al., 2019; Bense et al., 2023). Therefore, the experience of distress in these parents is not unexpected. A clinical trial study examining the effectiveness of a brief cognitive-behavioral intervention in reducing depression and anxiety in caregivers of children undergoing HSCT highlighted the importance of screening and identifying parents experiencing distress (Manne et al., 2016). This proactive approach can alert healthcare providers to implement effective interventions during critical periods in the lives of both parents and their children, preventing potential negative consequences of HSCT on their quality of life.

Based on the experiences of participants in the current study, a process of self-actualization unfolded for most of them. This self-actualization encompassed a transformation in life philosophy and the acquisition of new capabilities. In line with these findings, in a qualitative study involving parents from the anticipation stage to the completion of their child’s HSCT, participants consistently reported numerous positive outcomes despite facing significant difficulties. These benefits encompassed increased spirituality, gaining a new perspective on life, strengthening parent–child relationships, a greater appreciation for life, and a desire to help families whose children will undergo this process in the future (Schaefer et al., 2022). In another qualitative study, it was identified that parents of children receiving HSCT developed a desire to help others, felt empathy toward others, established stronger relationships with their spouses, family members, and acquaintances, and acquired newfound personal strength to endure life’s hardships (Beckmann et al., 2021). Furthermore, findings from a qualitative study in Sweden, focusing on parents who had passed four or more years after their child’s HSCT, revealed that HSCT significantly influenced the social and marital dynamics of parents. Some participants indicated that HSCT had a positive impact on their marital relationships, while others attributed their divorces to the challenges associated with HSCT (Forinder, 2004). Inconsistent with our findings, a study conducted by Rini et al. demonstrated that adverse life events, such as a child’s BMT, resulted in diminished benevolence, and declines in basic and spiritual beliefs, thereby hindering positive transformations in mothers (Rini et al., 2004). The concept of self-actualization in the findings of this study can be better explained by the theory of post-traumatic growth proposed by Tedeschi and Calhoun. According to their theory, a distressing and stressful event such as HSCT may trigger involuntary rumination and coping efforts. Over time, this rumination becomes more purposeful and intentional, leading to the development of a new worldview that allows parents to identify positive reappraisals of the HSCT experience (Tedeschi et al., 2018). Moreover, Muslims utilize their religious convictions to attain heightened spiritual states, and the Islamic cultural context diverges notably from other cultural contexts (Ebadi et al., 2009).

### 4.1 Limitations

This study’s limitations include the selection of participants from a referral hospital in an urban area of Iran, potentially impacting the transferability of our findings to other contexts and cultures. Hence, conducting similar research in diverse contexts, cultures, and countries is advisable. Furthermore, our study focused exclusively on parents of children with cancer undergoing HSCT. Future research could explore the experiences of parents whose children underwent HSCT for non-malignant diseases. Furthermore, future research employing longitudinal designs could offer a more comprehensive understanding of how parental experiences over time, considering variations between the early and late stages of treatment. Additionally, investigating the experiences of children undergoing HSCT would contribute valuable insights.

## 5 Conclusion

This study deepens our understanding of the experiences of parents with children undergoing HSCT. Following the cancer diagnosis and subsequent HSCT, parents undergo significant changes and encounter challenges affecting their physical, psychological, and social dimensions, creating a sense of being surrounded by hardships. Despite these difficulties, they also experienced a sense of self-actualization. Our findings underscore the importance for healthcare providers to be attentive to parents’ experiences throughout their child’s HSCT trajectory. Promoting open communication and offering guidance on addressing and coping with challenges is essential. This approach empowers parents to proactively express negative emotions, including anxiety, depression, and fear. Healthcare providers should emphasize the importance of parents articulating their concerns and feelings and seeking support from healthcare providers, family, and friends. The establishment of psychological support services in healthcare settings can facilitate personalized interventions to alleviate parents’ difficulties. Recognizing the positive aspects of parental experiences can contribute significantly to their overall well-being. By incorporating measures to assess self-actualization, healthcare providers can tailor interventions to support parents not only in coping with difficulties but also in fostering their strengths and positive experiences. Our study suggests several future research directions. Firstly, exploring the long-term effects of parental experiences during HSCT on their mental health and quality of life could provide valuable insights into the lasting impact of these challenges. Additionally, investigating the effectiveness of different support interventions, such as peer support groups or counseling services, in promoting resilience and enhancing parental well-being warrants further exploration. Furthermore, considering cultural and contextual factors in understanding parental experiences, particularly in diverse healthcare settings like Iran, could inform the development of culturally sensitive support programs.

## Data availability statement

The raw data supporting the conclusions of this article will be made available by the authors, without undue reservation.

## Ethics statement

The studies involving humans were approved by the study protocol received approval from the Ethics Committee affiliated with Tehran University of Medical Sciences (decree number: IR.TUMS.CHMC.REC.1401.145). Permissions to access participants’ information were obtained, and participants were informed about the study’s purpose, voluntary participation, data confidentiality, the right to withdraw at any point, and anonymity. Informed consent and permission to audio record interviews were obtained from participants before each interview. The studies were conducted in accordance with the local legislation and institutional requirements. The participants provided their written informed consent to participate in this study.

## Author contributions

MM: Conceptualization, Data curation, Formal analysis, Methodology, Validation, Writing – original draft, Writing – review & editing. NN: Conceptualization, Data curation, Formal analysis, Investigation, Methodology, Supervision, Validation, Writing – original draft, Writing – review & editing. AH: Conceptualization, Supervision, Writing – original draft, Writing – review & editing. BP: Conceptualization, Funding acquisition, Investigation, Methodology, Project administration, Supervision, Validation, Writing – original draft.
