# Supplementary material for: Parents’ experiences of living with a child with cancer undergoing hematopoietic stem cell transplantation: a qualitative content analysis study
Source: Front Psychol. 2024 Mar 12;15:1359978. doi: 10.3389/fpsyg.2024.1359978 (PMC10963479; doi:10.3389/fpsyg.2024.1359978)
Supplement: Supplementary file 1 [file Table_1.pdf]

Supplementary file 1. Participant's quotations in native language and English

Theme	Participant's quotations in English	Participant's quotations in native language (Persian)
**Surrounded by hardships**	“I am very worried about his future, and I don't know what will happen in the future. What if his illness relapses?” (Participant (P)8, Father).	من در مورد آینده او بسیار نگران هستم و نمی دانم در آینده چه اتفاقی خواهد افتاد. اگر بیماری او عود کند چه؟ (مشارکت‌کننده ۸، پدر)
	“Since we are using his own bone marrow, we are afraid of relapse. But those who receive it from someone else have a much better chance of recovery” (P9, Father).	از آنجایی که ما از مغز استخوان خودش استفاده می‌کنیم، از عود می‌ترسیم. اما کسانی که آن را از شخص دیگری دریافت می‌کنند، شانس بهبودی بسیار بیشتری دارند (مشارکت‌کننده ۹، پدر).
	"I kept telling myself that it's a relief that there's a suitable treatment for my child's illness and that he'll get better. I was very happy about that. But at the same time, I was worried because the transplant came with many challenges, and all of it was concerning to me" (P11, Mother).	من مدام به خودم می فتم که این باعث آرامش است که درمان مناسبی برای بیماری فرزندم وجود دارد و او بهتر می‌شود. از این بابت بسیار خوشحال بودم. اما در عین حال نگران بودم زیرا پیوند با چالش‌های زیادی همراه بود و همه این‌ها برای من نگران‌کننده بود (مشارکت‌کننده ۱۱، پدر).
	"I believe it was about two months after her transplant when they (physicians and nurses) mentioned she needed another cell injection. We were anxious because we thought her transplant had definitely been rejected" (P4, Mother).	من فکر می‌کنم حدود دو ماه پس از پیوند او بود که آن‌ها (پزشکان و پرستاران) اشاره کردند که او به تزریق سلول دیگری نیاز دارد. ما مضطرب بودیم زیرا فکر می‌کردیم پیوند او قطعاً رد شده است (مشارکت‌کننده ۴، مادر).
	"A few days after leaving the hospital, we suddenly noticed that my child's skin had become scaly. We were all concerned that his transplant might have been rejected again" (P3, Mother).	چند روز پس از ترخیص از بیمارستان، ناگهان متوجه شدیم که پوست فرزندم پوسته پوسته شده است. همه ما نگران بودیم که پیوند او دوباره رد شده است (مشارکت‌کننده ۳، مادر).
	"When I entered the transplant department, I felt intense fear and stress. Because two out of three friends who had their children undergo transplant before us tragically lost their kids during the transplant process. I was afraid of the possibility of losing my own child" (P10, Mother).	وقتی وارد بخش پیوند شدم، ترس و استرس شدیدی احساس کردم. چون از هر سه دوستی که قبل از ما فرزندان خود را تحت پیوند قرار داده بودند، دو نفر فرزندان خود را به‌طرز غم‌انگیزی در طی فرآیند پیوند از دست دادند. من از احتمال از دست دادن فرزندم می‌ترسیدم (مشارکت‌کننده ۱۰، مادر).
	"We were worried that since he was undergoing intense chemotherapy, if he were to contract COVID-19, we might lose him" (P9, Father).	ما نگران بودیم که از آنجایی که او تحت شیمی‌درمانی شدید قرار می‌گرفت، در صورت ابتلا به کووید ۱۹، ممکن است او را از دست بدهیم (مشارکت‌کننده ۹، پدر).

	"My daughter hasn't been able to attend school for two years now, so I'm concerned about her education. Additionally, I worry that if she grows up and wants to get married, she may face fertility issues" (P17, Mother).	دخترم دو سال است که نمی‌تواند به مدرسه برود، بنابراین من نگران تحصیل او هستم. علاوه بر این، نگران هستم که اگر او بزرگ شود و بخواهد ازدواج کند، ممکن است با مشکلات باروری مواجه شود (مشارکت‌کننده ۱۷، مادر).
	"To obtain a bone marrow sample, they (physician and nurses) administered her anesthesia and gave her an injection. My child was in pain. She cried while the cells were being extracted, and I was deeply upset to see her like that" (P18, Mother).	برای گرفتن نمونه مغز استخوان، آنها (پزشک و پرستاران) بیهوشی او را انجام دادند و به او تزریق کردند. بچه‌ام درد داشت. او در حین بیرون کشیدن سلول‌ها گریه کرد و من از دیدن او به شدت ناراحت شدم (مشارکت‌کننده ۱۸، مادر).
	"Prior to the transplant, he underwent a series of intense chemotherapy sessions, which were quite harsh. He experienced severe side effects, such as continuous vomiting of blood and darkening of the skin, which distressed me greatly" (P13, Mother).	قبل از پیوند، او تحت یک سری جلسات شیمی درمانی شدید قرار گرفت که بسیار سخت بود. او عوارض جانبی شدیدی مانند استفراغ مداوم خون و تیره شدن پوست را تجربه کرد که من را به شدت آزار می داد (مشارکت‌کننده ۱۳، مادر).
	"When those cells were injected, my mind was preoccupied with what the results of the samples would be. Every day, I anxiously awaited the test results for my child" (P5, Father).	زمانی که آن سلول ها تزریق شد، ذهن من درگیر این بود که نتایج نمونه ها چگونه خواهد بود. هر روز، مضطربانه منتظر نتیجه آزمایش فرزندم بودم (مشارکت‌کننده ۵، پدر).
	"I felt conflicted. On one hand, I was happy that my other child was a donor, but on the other hand, I was sad and afraid that this child would also face problems" (P6, Mother).	احساس تضاد داشتم. از یک طرف خوشحال بودم که فرزند دیگرم اهداکننده است، اما از طرف دیگر ناراحت بودم و می ترسیدم که این کودک نیز با مشکل مواجه شود (مشارکت‌کننده ۶، مادر).
	"I thought to myself, 'Well, my daughter has this problem. Now if we transplant my son's umbilical cord cells to her, what will happen if my son has a problem in the future and needs these cells?"(P12, Father).	با خودم فکر کردم خوب، دخترم این مشکل را دارد، حالا اگر سلول‌های بند ناف پسرم را به او پیوند بزنیم، اگر پسرم در آینده مشکلی داشته باشد و به این سلول‌ها نیاز داشته باشد، چه اتفاقی می‌افتد؟ (مشارکت‌کننده ۱۲، پدر).
	"All I worry about is my daughter, who stays in our own city. When my husband comes to visit us, she stays at home alone. I am a mother, and I always worry about her" (P3, Mother).	تمام نگرانی من دخترم است که در شهر خودمان می‌ماند. وقتی شوهرم به دیدن ما می‌آید، او تنها در خانه می‌ماند. من یک مادر هستم و همیشه نگران او هستم (مشارکت‌کننده ۳، مادر).
	"We came from our city to the capital for the transplant, and I didn't go back for five years and I didn't see our relatives, and it was very difficult for me to be away from my family" (P10, Mother)	ما از شهرمان برای پیوند به پایتخت آمدیم و پنج سال است که برنگشتیم و اقواممان را ندیدم و دوری از خانواده برایم بسیار سخت بود (مشارکت-کننده ۱۰، مادر).

	"I didn't have any problems before my son's transplant, but after that, I experienced insomnia, hypertension, and hypothyroidism"(P20, Mother).	قبل از پیوند پسرم هیچ مشکلی نداشتم، اما بعد از آن دچار بی‌خوابی، فشار خون بالا و کم‌کاری تیروئید شدم (مشارکت‌کننده ۲۰، مادر).
	"There were days when I even contemplated suicide. I would tell myself it was my fault. I was stressed, I was greedy, and I thought somehow that I was the cause of my child's illness" (P10, Mother).	روزهایی بود که حتی به خودکشی فکر می‌کردم. به خودم می‌گفتم این تقصیر من است. استرس داشتم، حرص می‌خوردم و به نوعی فکر می‌کردم که علت بیماری فرزندم هستم (مشارکت‌کننده ۱۰، مادر).
**Self-Actualization**	"I used to be quite sensitive, but not anymore. Now, I realize that life can change and end in an instant for anyone, so I've learned to stay calm" (P5, Father).	"قبلاً کاملاً حساس بودم، اما دیگر نه. اکنون، می‌دانم که زندگی می‌تواند در یک لحظه برای هر کسی تغییر کند و به پایان برسد، بنابراین یاد گرفته‌ام که آرام بمانم (مشارکت‌کننده ۵، پدر).
	"After the transplant, I've come to appreciate my child's worth even more. I've gained a deeper understanding of the value of life; I believe these challenges are necessary for us to truly appreciate life" (P20, Mother).	بعد از پیوند، ارزش فرزندم را حتی بیشتر می‌دانم. درک عمیق‌تری از ارزش زندگی به دست آورده‌ام؛ معتقدم این چالش‌ها برای ما ضروری هستند که واقعاً زندگی را قدر بدانیم (مشارکت‌کننده ۲۰، مادر)
	"We used to provide minimal assistance to this (NGO) institution, but now, after my child's transplant, we contribute more. We've come to realize how much it truly helps the patients. They pay for the medicine and hotels for people/families traveling from distant/other cities, and provide many other aids" (P6, Mother).	ما قبلاً کمک کمتری را به این مؤسسه (NGO) ارائه می‌کردیم، اما اکنون، پس از پیوند فرزندم، بیشتر کمک می‌کنیم. ما متوجه شده‌ایم که واقعاً چقدر به بیماران کمک می‌کند. آنها هزینه دارو و هتل‌ها را برای مردم/ خانواده‌هایی که از شهرهای دور/دیگر سفر می‌کنند، می‌پردازند و بسیاری از کمک‌های دیگر را ارائه می‌کنند (مشارکت‌کننده ۶، مادر).
	"After my son's transplant, I handed over the test results of myself, my wife, and my children to the hospital staff responsible for finding donors. I told them to contact us if our test results matched those of a child in need of a transplant" (P19, Father).	پس از پیوند پسرم، من نتایج آزمایش خودم، همسرم و فرزندانم را به پرسنل بیمارستانی که مسئول یافتن اهداکنندگان بودند، تحویل دادم. به آنها گفتم اگر نتایج آزمایش ما با یک کودک نیازمند پیوند مطابقت داشت با ما تماس بگیرند. (مشارکت‌کننده ۱۹، پدر).
	"After the transplant, I felt a newfound strength within me. Now, no matter what challenges come my way, I'm determined to fight until the end" (P5, Father).	بعد از پیوند، نیروی تازه‌ای را در درون خود احساس کردم. اکنون، مهم نیست که چه چالش‌هایی بر سر راهم قرار می‌گیرد، مصمم هستم که تا آخر بجنگم (مشارکت‌کننده ۵، پدر)

	"With my child's transplant, I've gained valuable experience about the disease and the transplant process, enabling me to offer help and guidance to people in need" (P15, Father).	با پیوند فرزندم، تجربیات ارزشمندی در مورد بیماری و فرآیند پیوند به دست آوردم، که به من این امکان را می‌دهد تا به افراد نیازمند کمک و راهنمایی کنم (مشارکت‌کننده ۱۵، پدر).
	"I was divorced from my husband, but due to my daughter's plea, urging me to reconcile with her father for the sake of her illness, I had to remarry him in order to facilitate my daughter's treatment. This circumstance led my husband and me to live together again" (P17, Mother).	من از شوهرم طلاق گرفته بودم اما به دلیل درخواست دخترم مبنی بر اینکه به خاطر بیماری با پدرش آشتی کنم مجبور شدم برای تسهیل روند درمان دخترم دوباره با او ازدواج کنم که همین موضوع باعث شد من و شوهرم دوباره با هم زندگی کنیم (مشارکت‌کننده ۱۷، مادر).
	"After the transplantation, I concluded that the only constant companion of a person is God, and my faith in God has multiplied. I feel that God is more attentive to us now" (P11, Mother).	بعد از پیوند به این نتیجه رسیدم که تنها همراه همیشگی انسان خداست و ایمانم به خدا چند برابر شده است. احساس می‌کنم اکنون خدا بیشتر حواسش به ماست (مشارکت‌کننده ۱۱، مادر).
	"My child's disease was a test from God, and I felt God in every moment of my child's disease and transplantation" (P12, Father).	بیماری فرزندم آزمایشی از جانب خدا بود و من در هر لحظه از بیماری و پیوند فرزندم، خدا را احساس می‌کردم (مشارکت‌کننده ۱۲، پدر).
